# Epigenetic regulation of osteopontin splicing isoform c defines its role as a microenvironmental factor to promote the survival of colon cancer cells from 5-FU treatment

**DOI:** 10.1186/s12935-020-01541-z

**Published:** 2020-09-14

**Authors:** Siyuan Chang, Jing Huang, Huan Niu, Jing Wang, Yang Si, Zhigang Bai, Shan Cheng, Wei Ding

**Affiliations:** 1 Department of Medical Genetics and Developmental Biology, School of Basic Medical Sciences, Capital Medical University, Beijing, 100069 China; 2 Department of General Surgery, Beijing Friendship Hospital, Capital Medical University, Beijing, 100050 People’s Republic of China

**Keywords:** OPN, Splicing isoforms, Cell survival, Nuclear calcium, MeCP2

## Abstract

**Background:**

Drug resistance to 5-fluorouracil (5-FU) and recurrence after chemotherapy in colorectal cancer remain a challenge to be resolved for the improvement of patient outcomes. It is recognized that a variety of secretory proteins released from the tumor cells exposed to chemo-drugs into the tumor microenvironment (TME) contributed to the cell-to-cell communication, and altered the drug sensitivity. One of these important factors is osteopontin (OPN), which exists in several functional forms from alternative splicing and post-translational processing. In colon cancer cells, increased total OPN expression was observed during the progression of tumors, however, the exact role and regulation of the OPN splicing isoforms was not well understood.

**Methods:**

We assayed precisely the abundance of major OPN splicing isoforms under 5-FU treatments in colon cancer cell lines with different sensitivities to 5-FU, and also evaluated the effects of the condition medium from OPN splicing isoforms overexpressed cells on cell functions. The methods of nuclear calcium reporter assays and ChIP (chromatin immunoprecipitation) assays were used to investigate the molecular mechanism underlining the production of OPN isoforms.

**Results:**

We discovered that OPNc was a most increased splicing isoform to a significant abundance following 5-FU treatment of colon cancer cells. OPNc as a secretory protein in the conditioned medium exerted a more potent effect to promote cell survival in 5-FU than other OPN isoforms. The kinetic response of nuclear calcium signals could be used to indicate an immediate effect of the conditioned medium containing OPNc and other isoforms. Methyl-CpG binding protein 2 (MeCP2) was identified to regulate the splicing of *opn* gene, where the phosphorylation of MeCP2 at S421 site, possibly by calmodulin dependent protein kinase II (CaMKII) was required.

**Conclusions:**

The results demonstrated that the production of OPNc was highly controlled under epigenetic regulations, where MeCP2 and the activation of nuclear calcium signaling were involved. It was also suggested that OPNc could transmit the stress signal of cells upon chemotherapy in TME and promoted the survival of adjacent colon cancer cells.

## Background

Currently, chemotherapy remained to be a major procedure for the treatment of colorectal cancers. Accumulating evidences from various clinical or animal studies implied that chemotherapy could stimulate drug resistance even at early phases during the treatment and turn out to promote tumor growth and metastasis [1, 2]. Chemotherapeutic agent, 5-fluorouracil (5-FU) and its derivatives, is a conventional constituent, widely used for the treatment of multiple types of tumors, including colorectal cancer (CRC). Despite 5-FU was able to quickly reduce the tumor burden following drug administration by inducing cell apoptosis, the increased levels of spontaneous apoptotic cell death in the tumor mass did not necessarily associated with better prognosis as observed in CRC patients. Sometimes, patients received adjuvant 5-FU exhibited shorter overall survival compared with patients who subjected to only surgery operations, especially in cases of CRC reoccurrence [3]. From an increasing number of reports, it strongly indicated that microenvironmental factors secreted from cancer cells exposed to chemotherapy agents directly stimulated and were critically involved in the development of drug resistance through autocrine and paracrine mechanisms [4]. The identification or depletion of such factors was suggested as an important approach to validate potential biomarkers for prognosis, or screening for therapeutic targets when resistance was being developed.

Among various tumor secreted molecules, osteopontin (OPN) was previous reported to associate with cancer progression and drug resistance in CRC and other cancer types [5]. OPN is a multifunctional secreted glycoprotein implicated in diverse physiopathological processes required for tumor formation and progression, such as cell proliferation, inhibition of apoptosis, invasion and metastasis, angiogenesis and chemo-resistance [6]. From laboratory experiments in cancer cells, overexpression of OPN has been found to render the resistance to cytotoxic drug induced apoptosis and accelerated tumor progression and metastasis. Up regulation of OPN at both of the protein and mRNA levels were found in a wide range of advanced cancers from clinical reports [7].

In fact, the OPN transcripts containat least three major species of OPN splicing isoforms (OPN-SI), named OPNa, OPNb and OPNc respectively. OPNa is regarded as the full-length wild type form. OPNb and OPNc are the mutually excluded splicing isoforms, where the former lacks of exon 5 and the latter is without exon 4 [8]. The precise regulation and exact functions of different OPN-SIs remained to be obscure, except from recent reports where certain OPN-SI was suggested to be more potent in promoting the development of tumor malignant phenotypes [9, 10]. Among the known OPN-SIs, OPNc was revealed with most importance of clinical values. OPNc was upregulated in ovarian, breast and pancreatic cancers, which correlated with the histological grade and TNM stage, and well predicted tumor recurrence or metastasis [11–13]. In CRC patients, OPN protein levels were increased in both plasma and tissues, which in negative correlation with the survival [14, 15]. However, the roles of OPN-SIs, as well as the change in levels following 5-FU treatments were not fully characterized. To better understand the importance of a specific isoform of OPN splicing isoforms, several questions need to be concerned for conducting in vitro experiments. It will be informative to obtain the answers for whether a specific OPN-SI is more potent to render the malignant cellular phenotype, and which of cytoplasmic or secreted protein forms contributes more to define the biological function of an OPN-SI. It is also important to address the issues on how the production of OPN-SIs is regulated, and/or how the OPN-SI functions are transmitted and propagated, especially in context of drug responsiveness or resistance. In-depth investigation on OPN-SIs will expand the knowledge to cancer cell biology and could potentially lead to the validation of certain species as applicable cancer biomarkers.

In the present study, we found that exposure in 5-FU resulted in significant changes in the cellular abundance of OPN-SIs, and the application of OPN-SIs as in the secretory forms in the conditioned medium influenced the cell sensitivity to 5-FU. It was shown that the increase of OPNc, which could promote the cell survival through transmitting stress signal to adjacent cells in tumor microenvironment (TME), required the activation of nuclear calcium signals and the subsequent modulation of epigenetic regulator, Methyl-CpG binding protein 2 (MeCP2). Our finding provided an important piece of evidence to demonstrate how epigenetic regulation of splicing products to mediate the adaptation of cancer cells to stress environment and transmit to preemptive phenotype morphing signals to adjacent partners.

## Materials and methods

### Tissue culture and cell treatments

The human colon cancer cell lines of HT115and HCT-8 were obtained from European Collection of Animal Cell Cultures (ECACC) (Salisbury, UK) and American Type Culture Collection (ATCC) (Manassas, VA, USA), respectively. HT115 was maintained at 37 °C in a 5% CO_2_ incubator in Dulbecco’s modified Eagle’s medium (DMEM) with 15% fetal bovine serum (FBS) (Biological Industries, Kibbutz BeitHaemek, Israel) and 1% penicillin–streptomycin (Keygen Biotech, Nanjing, China). HCT-8 was maintained in RPMI-1640 medium with 10% FBS. Research grade 5-fluorouracil (5-FU) (MCE, Monmouth Junction, NJ, USA), KN-93 (an inhibitor to CaMKII, calmodulin dependent protein kinase II) (MCE), and the epigenetic modifier of 5-Aza-2′-deoxycytidine (Sigma-Aldrich, St. Louis, MO, USA) known to cause hypomethylation of DNAs were used in the assays to evaluate cell survival and gene expression responsive to nuclear calcium signal or DNA methylation related genome remodeling. The OPN antibody (8448, Abcam, Cambridge, MA, USA) was used in the in vitro antibody neutralization studies.

### Preparation of the conditioned mediums (CMs) containing OPN-SIs

A number of 1.0 × 10^5^ cells were seeded in 60 mm plates. After transfected with the plasmids carrying the expression cassette of OPNa, OPNb and OPNc for 6 h, cells were cultured in 2 ml fresh culture medium with FBS for 48 h. The supernatant culture mediums were collected and freeze concentrated to 100 μl using Lyophilizer (Alpha 1-2LDplus, Martin Christ, Osterode am Harz, Lower Saxony, Germany). In assays, 10 μl CM was added to 1 ml fresh medium for conditioned culture.

### Human colorectal tumor specimen

The ethical approval for the present study was obtained from the Ethics Committee of Beijing Friendship Hospital. A total of 325 colorectal cancer tissues and 193 adjacent paracancerous tissues were collected from patients who received curative resection at Beijing Friendship Hospital. The tissue samples were processed immediately after surgical operations following the SOP and registered into the Tissue Bank of Cancer Institute of Capital Medical University. The clinic-pathological information, including patient age, gender, as well as tumor anatomical site, TNM stage and differentiation grade was recorded for documentation.

### Vector preparation and transfection

The expression plasmids of FLAG tagged OPN-SIs (OPNa, OPNb and OPNc) were cloned individually into pENTER vectors by Vigene Bioscience Co., Ltd. (Shandong, China). The pcDNA3.1-nuGCaMP6s-tdTomato plasmid was generously transferred from Dr. H Ye [16]. The siRNAs targeting MeCP2, as listed in Table 1, were purchased from GenePharma (Shanghai, China). The cells were transfected with either the OPN plasmids or paired siRNA oligos using a Lipofectamine™RNAi^MAX^ Kit (Invitrogen, Waltham, Massachusetts, USA) following the vendor’s recommended protocols. Western blotting was performed to detect the protein levels of the corresponded genes at 48 h post transfection.

**Table 1:** The primers and siRNAs used in this study

	Target	Oligonucleotide sequence
qPCR	OPN-t	F: GCCGAGGTGATAGTGTGGTT
		R: AACGGGGATGGCCTTGTATG
	OPN-a	F: GCCGAGGTGATAGTGTGGTT
		R: AACGGGGATGGCCTTGTATG
	OPN-b	F: ATCTCCTAGCCCCACAGAC
		R: AAAATCAGTGACCAGTTCATCAG
	OPN-c	F: TGAGGAAAAGCAGAATGCTG
		R: GTCAATGGAGTCCTGGCTGT
	OPN-exon4	F: AAGATAGCCACACTCAGGCCATTTG
		R: CAGCCTCATCTGTGGATGGCTTAAC
	OPN-exon5	F: AACAAGAGGTAAGTTCTCATTTTCA
		R: AACTTATCCGAGGAAACCTAGTATT
	HAUS8	F: ACAGGGTGCCACCTCTTTCT
		R: TGCAGGTGCTGGACTTACTG
	GAPDH	F: GGAGCGAGATCCCTCCAAAAT
		R: GGCTGTTGTCATACTTCTCATGG
siRNA	Scramble	Sense: UUCUCCGAACGUGUCACGUTT
		Antisense: ACGUGACACGUUCGGAGAATT
	MeCP2-1	Sense: GCUUAAGCAAAGGAAAUCUTT
		Antisense: AGAUUUCCUUUGCUUAAGCTT
	MeCP2-2	Sense: GCUUCCCGAUUAACUGAAATT
		Antisense: UUUCAGUUAAUCGGGAAGCTT

### RT-qPCR assays

Total RNA was isolated using Trizol (Life Technologies, Carlsbad, CA, USA). HiScript II Q RT Kit (Vazyme, Nanjing, China) was used for reverse transcription. NovoStart^®^ SYBR qPCRSuperMix Plus (Novoprotein, Shanghai, China) was used to quantify gene expression level from the obtained cDNA. The primers for detecting OPNt, OPNa, OPNb and OPNc are listed in Table 1. GAPDH was used as the loading reference.

### Western blotting

Western blot analyses were performed as earlier described [9]. Briefly, samples of cell lysates were prepared and separated by 10% SDS-polyacrylamide gel electrophoresis (SDS-PAGE), then transferred onto polyvinylidene fluoride (PVDF) filters. The probing antibodies were against the following antigens: MeCP2 (3456, Cell Signaling Technology, Danvers, MA, USA), pMeCP2 (S421) (AP3693a, Abgent, San Diego, CA, USA) and GAPDH (TA-08, ZSGB-BIO, Beijing, China).

### Cell viability assay

A number of 5000 cells were seeded in each well of 96-well plates and cultured with 5-FU at 0, 1, 5, 10, 20 and 40 μg/ml for 48 h. The cell viability was determined using cell counting kit (CCK8) (KeyGEN BioTECH, Jiangsu, China) according to the vendor’s standard protocols. The plates were scanned at 450 nm for absorbance using a spectrophotometer (BioTek, Winooski, VT, USA). Each data point was measured for the average from six duplicates. The experiments were repeated independently for 3 times.

### Apoptosis assay

Cells were seeded in 6-well plates at 1.0 × 10^5^ cells/well and treated with 5-FU at 20 μg/ml for 24 h, except the control group where dimethyl sulfoxide (DMSO) was applied as the vehicle used to dilute drugs. The cell apoptosis was assayed using an Annexin V-FITC Apoptosis Detection Kit (KeyGEN BioTECH, Jiangsu, China) following the manufacturer’s standard protocol.

### Immunofluorescence

Cells of 1.0 × 10^5^ were plated onto a glass coverslip placed into the well of a 6-well plate. The cells on coverslips were fixed, permeabilized, blocked and washed with phosphate-buffered saline (PBS). Anti-γH2AX (05-636, Merck Millipore, Darmstadt, Germany) was used as the primary antibody and an Alexa Fluor^®^ 488 secondary antibody (Life Technologies, MA, USA) was used for incubation in the dark. The nuclei were stained with Hoechst 33258 (Sigma-Aldrich, St. Louis, MO, USA) prior to the examination and image acquisition under a confocal system (Leica Microsystems TCS SP8. Wetzlar, Germany). Control samples without adding the primary antibody were prepared for determining the level of non-specific noise.

### Cellular calcium imaging

Cells cultured on coverglass were transfected with pcDNA3.1-nuGCaMP6s-tdTomato. Live confocal microscopy was performed at 24 h post transfection using an UltraVIEWVoX system (PerkinElmer, Waltham, MA, USA). Fluorescent signals from two fluorophores (GCaMP6s and tdTomato) were collected at 488 nm/543 nm for excitation and 493-552 nm/560-605 nm for emission. Images were acquired by scanning in the frame scan mode (512 pixels, 1 s/frame). The fluorescence intensity and the ratio of GCaMP6s to tdTomato channels (expressed as G/R) were calculated and plotted using a custom macro script for Image J1.50i (National Institutes of Health, Bethesda, MD, USA).

### Chromatin immunoprecipitation (ChIP) assay

Cells were incubated in 1% formaldehyde for crosslinking. Soluble sheared chromatin (DNA fragments of 200-500 bp in average) was obtained by sonication on ice (45% Pw, 1 s on/1 s off, 15 s). The sample was subjected for immunoprecipitation except the saved 1% used as the input. Protein A/G PLUS-Agarose (sc-2003, Santa Cruz Biotechnology, Dallas, Texas, USA) were loaded either with the following ChIP-validated anti-MeCP2 (ab2828, Abcam, Cambridge, UK) antibody or control IgG (2729, Cell Signaling Technology). The beads were incubated with chromatin extracts at 4 °C overnight with a rotation shaker. The immunoprecipitated DNA was purified using DNA isolation kit (TaKaRa, Kusatsu, Shiga, Japan). Quantification of ChIP-enriched DNA was quantified by real-time PCR with specific primers (Table 1). The enrichment ratio was determined according to the following formula: (% IP/INPUT = 2^[(Ct (x% input) − log (x %)/log2) − Ct (IP)] × 100^).

### Immunohistochemistry (IHC)

The colorectal tissue sections (5 μm thick) were deparaffinized, rehydrated, rinsed with ddH_2_O, and washed with Tris-buffered saline (TBS). IHC staining was carried out using an automated Ventana BenchMark GX instrument (Ventana Medical Systems, Inc., Tucson, AZ, USA). Probesof anti-MeCP2 (3456, Cell Signaling Technology) and anti-pMeCP2 antibody (AP3693a, Abgent), and a 5mC antibody (AMM99021, AVIVA, San Diego, CA, USA) was used. The intensity of staining was determined using ImageJ1.50i. Randomly selected 10 microscopic fields were subjected to semi-quantification from computer assisted image analyses.

### Statistical analysis

The association between gene expression and clinical factors was analyzed using Mann–Whitney U test. The analysis of variance (ANOVA) was used to determine the statistical significance of data in multiple groups. The Student’s t-test was used to compare cell functions between paired groups. Cases of *p*-value < 0.05 was defined as statistically significant. The program of Prism 8 (GraphPad Software, Inc., La Jolla, CA, USA) was used for data plotting.

## Results

### The OPNc mRNA levels were preferentially upregulated in colon cancer cells following short 5-FU treatment

**Fig. 1:**
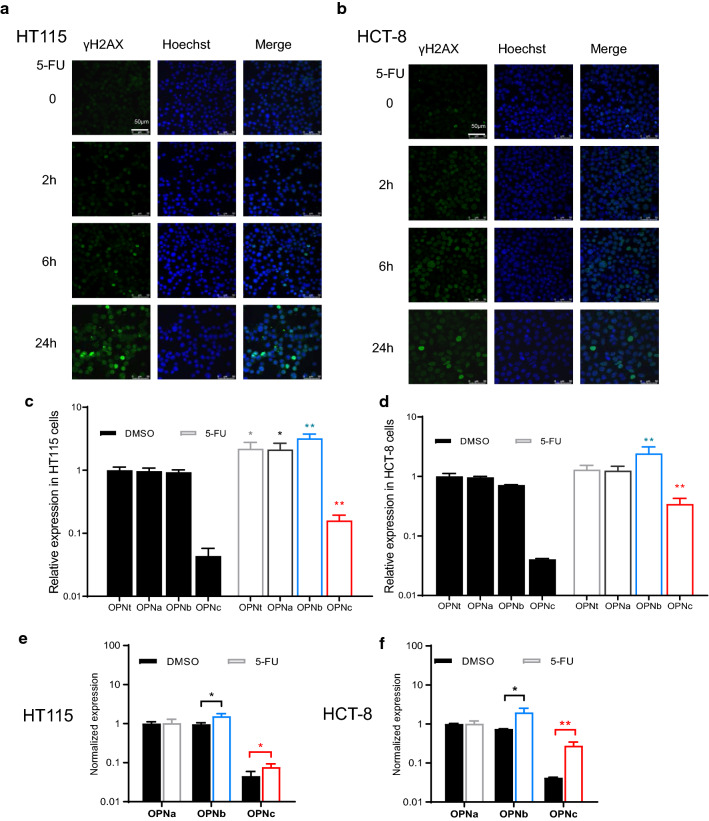
OPN splicing isoform c was selectively upregulated in colon cancer cells briefly treated with 5-FU. HT115 (**a**) and HCT-8 (**b**) cells treated with 20 μg/ml 5-FU for 2, 6 or 24 h, and then stained for γH2AX. The mRNA levels of OPN splicing isoforms in HT115 (**c**) and HCT-8 (**d**) cells exposed to 6 μg/ml 5-FU for 40 min. Normalized OPN-SI expression for relative abundance. (n = 3, *p < 0.05, **p < 0.01)

The treatments of 5-FU in colon cancer cells was known to introduce DNA damages which could be indicated by increased staining of γH2AX as a quick response molecule of DNA repair activation, prior to the induction of cell apoptosis. Comparing HT115 and HCT-8 cells exposed to 20 μg/ml 5-FU at 2, 6 and 24 h (Fig. 1a, b), HT115 cells was shown to be more sensitive to 5-FU treatments. Yet, the significant accumulation of damaged DNAs with double strand breaks was not observed within a period of 2 h. However, even when exposed to 5-FU for as short as 40 min, the significant increase in OPN expression, as well as each of its major splicing isoforms of OPNa, OPNb and OPNc, were detected in both cell lines (Fig. 1c, d) as determined by RT-qPCR assays. To better characterize the relative abundances of OPNb and OPNc, normalized values in ratio of OPNb/OPNc to OPNt were calculated, where a 1.6-fold/1.7-fold increase in HT115 cells (Fig. 1e) and a 2.7-fold/6.9-fold in HCT-8 cells (Fig. 1f) were shown with statistical significance. These data indicated that the production of alternative splicing isoforms of *opn* in mRNA forms appeared to be rapid events following short 5-FU treatments. Among the major splicing isoforms, OPNc was the most sensitive indicator in response to 5-FU treatment in colon cancer cells.

### Conditioned medium collected from OPNc overexpressed cells significantly reduced the sensitivity to 5-FU in HT115 and HCT-8 cells

**Fig. 2:**
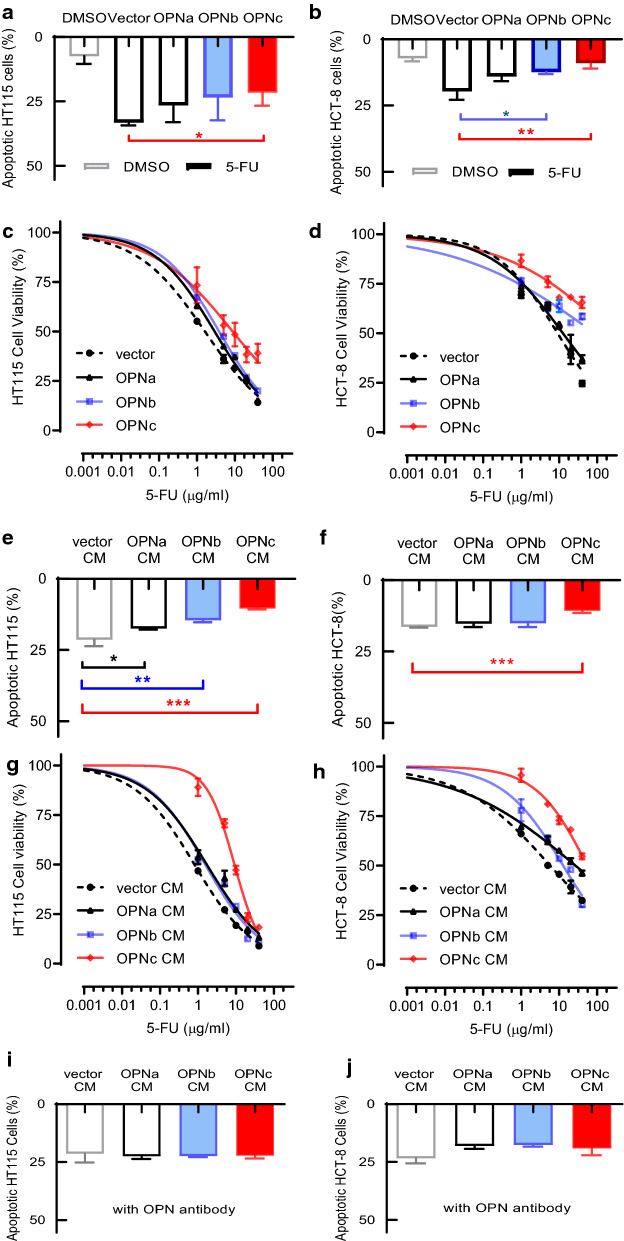
Overexpressed OPNc in secreted forms significantly reduced the sensitivity to 5-FU in both HT115 and HCT-8 cells. Rate of apoptosis in HT115 (**a**) and HCT-8 (**b**) cells transfected with OPN-SIs following 5-FU (20 μg/ml) treatments for 24 h as determined by flow cytometry. **c**, **d** Survival of transfected cells following 5-FU treatments (0, 1, 5, 10, 20 and 40 μg/ml for 48 h) as determined by CCK8 assays. **e**, **f** Cells cultured in conditioned medium (CM) from of OPN-SIs overexpressed cells and treated with 20 μg/ml 5-FU for 24 h for apoptosis assays. Cell survival in HT115 (**g**) and HCT-8 (**h**) subjected to dose-dependent response of 5-FU in OPN-SIs CMs. **i**, **j** Apoptosis assays using CMs with addition of 20 μg/ml OPN-neutralizing antibody. Data were represented as the mean ± SD from 3 (apoptosis) or 6 (viability) independent experiments. *p < 0.05, **p < 0.01, ***p < 0.001

OPN proteins were shown to influence the sensitivity of several anti-cancer drugs as previously reported in hepatocellular carcinoma and glioma cells [17]. To find out whether individual OPN-SI exerts a similar effect in colon cancer cells, we transfected OPNa, OPNb and OPNc with FLAG tags into HT115 or HCT-8 cells (Additional file 1: Fig. S1a, b), then performed apoptosis and cell viability assays under different concentration of 5-FU with transfected cells. As shown in Fig. 2a, b, overexpression of OPNa, OPNb or OPNc indeed reduced the rate of apoptosis by flow cytometry following 20 μg/ml 5-FU treatments for 24 h, from 33% to 27%, 23% and 21% in HT115 cells. Similar results were obtained from experiments in HCT-8 cells, where OPNc displayed a most significant effect. We also examined the transfected cells for the sensitivity to the 5-FU dose administration curves (Fig. 2c, d). The data also indicated that OPNc was the most potent form to promote cell survivals in the presence of increase 5-FU concentrations.

As OPN of its splicing isoforms may exist as secretory proteins (Additional file 1: Fig. S1c, d), we next prepared the conditioned medium (CM) from the culture of OPN-SIs transfected HT115 and HCT-8 cells. By applying the obtained CMs on HT115 or HCT-8 cells, the apoptosis and sensitivity to 5-FU were re-evaluated (Fig. 2e–h). As a striking surprise, the OPNc-CM exerted a much stronger effect to reduce the sensitivity of HT115 and HCT-8 cells to 5-FU than in colon cancer cells transfected with OPNc. To examine whether such effect was indeed rendered by the OPN protein in the CM, we used an OPN antibody to deplete the soluble OPN-SI from the CM prior to the application to cell cultures. We found that the OPN antibody neutralization significantly depleted the effects on drug sensitivity (Fig. 2i, j).

### OPN-SIs provided in CMs induced robust activation of nuclear calcium signal and altered OPN splicing in return

**Fig. 3:**
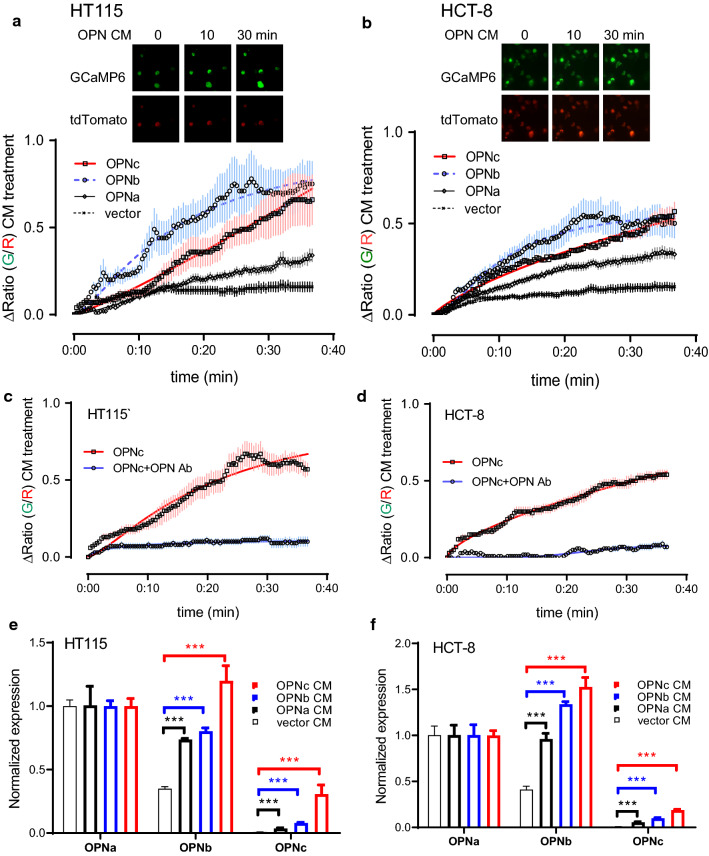
Conditioned medium from OPNc overexpressed cells induced cellular levels of OPN-SIs dependent to the activation of nuclear calcium signals. Nuclear Ca^2+^ in HT115 (**a**) or HCT-8 (**b**) cells transfected with nuGCaMP6s-tdTomato plasmid and stimulated with OPN-CMs from 0 to 40 min with representative live fluorescence images during assays. A total of 10 to 30 individual cells were selected for quantitative analyses of the ratio between GCaMP6s and tdTomato fluorescence intensities (lower). **c**, **d** Quantitative analyses in cells induced by OPNc-CM and CM with addition of OPN-neutralizing antibody. The normalised mRNA levels of *opn* splicing isoforms in HT115 (**e**) and HCT-8 (**f**) cells as determined by quantitative RT-qPCR after exposure in OPN-CMs for 40 min

Considering the recent discovery of cellular calcium as a regulator to a number of signaling pathways responsible for gene transcription and pre-mRNA splicing besides it function as a critical second messenger for cell survival [18], we took the advantage of a GCaMP based reporter and used it to detect the changes in nuclear calcium concentration following treatment with OPN-CMs. From the results shown in Fig. 3a, b, all CMs containing OPN-SIs were able to elicit robust responses in nuclear calcium signals. Interestingly, the kinetics of Ca^2+^ rising was different between HT115 and HCT-8 cell lines. Also, OPNc-CM and OPNb-CM appeared to give faster responses as compared to cases of OPNa, which consistently indicated that OPNc could be a more functional isoform to rapidly respond from stress conditions, such as 5-FU treatments. When the OPNc-CM was neutralized by OPN antibody, the nuclear calcium signal was significantly reduced (Fig. 3c, d). This suggested that cells exposed to secreted OPNc could directly trigger a responsive activation of nuclear calcium signals. Given the facts that OPN isoforms are the important molecules in calcium metabolism and involved in inflammatory responses in various tissues, and calcium response is a physiological process for cells to adapt to stress environments, our findings on OPN-SIs functions to trigger immediate nuclear calcium signaling changes could be of importance in other non-cancer related studies. With the rapid transmission of calcium signals, secretory proteins (such as OPNc) as microenvironmental factors can easily propagate the cell stress signal to adjacent cells, and may also remodel the cells with preemptive capacities for adaptation.

To address whether the expression of OPN splicing isoforms was altered by the stimulation of OPN-CMs, we detected the mRNA levels of OPN-SIs in colon cancer cells. The increased mRNA levels of OPNa, OPNb and OPNc were observed in the OPN-CMs treated HT115 and HCT-8 cells (Fig. 3e, f), in comparison to those in cells treated with vector control CM. Among three OPN-CMs, OPNc-CM played a stronger role than OPNa and OPNb in promoting the generation of OPN splicing isoforms, especially OPNc. These data suggested that OPNc could be a dominant isoform of OPN in colon cancer cells, generated and secreted by tumor cells exposed to cytotoxic therapy. The secreted OPNc transmitted the stress signals of tumor cells and stimulated the OPNc generation and secretion in adjacent cells, which could play as a messenger to spread the stress signals and contribute to cell survival in a positive feedback manner in TME.

### Inhibitor to either calcium-dependent kinase or DNA methylase attenuated the pro-survival effects of OPNc in 5-FU treated cells

**Fig. 4:**
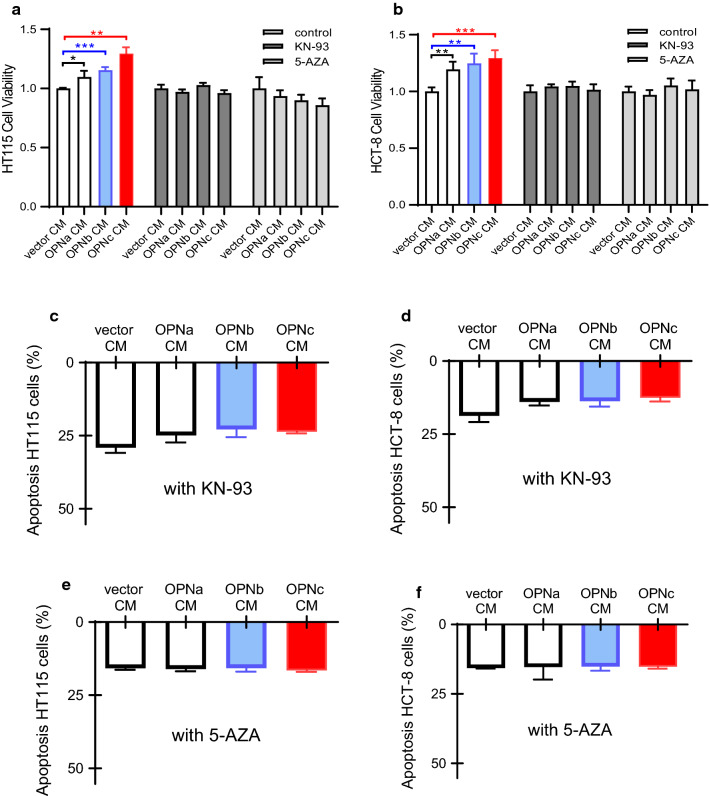
Inhibition of CaMKII or methylase activities attenuated the anti-apoptotic effects of CMs with OPN-SIs. The cell survival from CCK8 assays in HT115 (**a**) and HCT-8 (**b**) cells preadminstered with 5 μM KN-93 or 5 μg/ml 5-AZA and subjected to 6 μg/ml 5-FU in various OPN-SI CMs (n = 6). **c**–**f** Apoptosis assays in HT115 and HCT-8 cells by flow cytometry under conditions described as in (**a**) and (**b**) with 20 μg/ml 5-FU were shown in (**c**–**f**) (n = 3).The data were represent the mean values ± SD. *p < 0.05, **p < 0.01, ***p < 0.001

CaMKII is a serine/threonine-specific protein kinase that is regulated by the Ca^2+^/calmodulin complex. CaMKII is involved in many signaling cascades and is thought to be an important mediator of learning and memory [19]. Recently, it was also reported to be important for tumor cell growth, cell cycle regulation and cell differentiation [20]. In the present study, when the activity of CaMKII was inhibited by the specific inhibitor, KN-93, the OPN-CMs increased cell survivals from 5-FU treatment was attenuated (Fig. 4a, b). The similar results were also observed in cell apoptosis assays. The OPN-CMs attenuated cell apoptosis were depleted both in HT115 and HCT-8 cells (Fig. 4c, d).

Nuclear Ca^2+^ signaling in cells could affect cell survival through regulating gene transcription and pre-mRNA splicing for several signaling pathways. The status of DNA methylation also modulates gene transcription and pre-mRNA alternative splicing. Here, we also evaluated effects of methylase inhibitor on OPN-CMs promoted cell survivals. Results showed that treatment of methylase inhibitor, 5-AZA, depleted the OPN-CMs increased cell survival of HT115 and HCT-8 cells from the 5-FU treatment both in cell viability assays (Fig. 4a, b) and in apoptosis investigations (Fig. 4e, f). These results suggested that the activity of CaMKII and the status of DNA methylation are important in the OPN-CMs regulating cell sensitivity to 5-FU.

### MeCP2 with S421 phosphorylation was responsible for the regulation of OPN alternative splicing in colon cancer cells

**Fig. 5:**
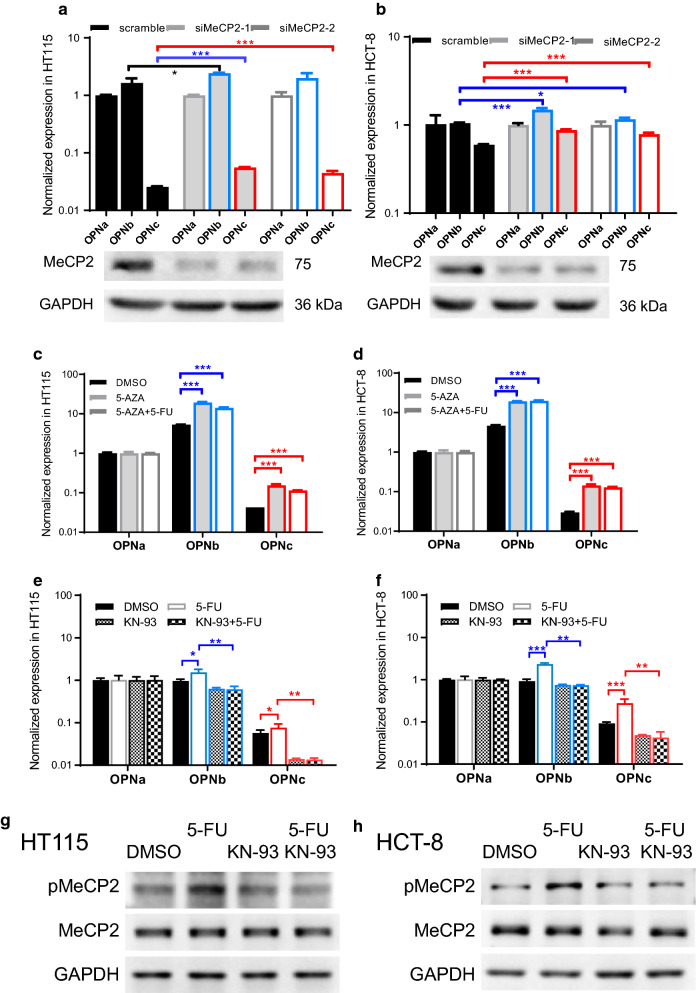
Enhanced splicing for the production of OPN-SIs was dependent to the phosphorylation of MeCP2. The mRNA levels of OPN-SIs in HT115 (**a**) and HCT-8 (**b**) cells transfected with MeCP2 siRNAs. The effect of 5-AZA on 5-FU induced OPN splicing in HT115 (**c**) and HCT-8 (**d**) cells. Cells were treated with 5 μg/ml 5-AZA or 5 μg/ml 5-AZA and 6 μg/ml 5-FU for 24 h. The OPN-SIs mRNA levels in HT115 (**e**) and HCT-8 (**f**) cells in the present of 6 μg/ml 5-FU or 5 μM KN-93 or both for 24 h. The protein levels of MeCP2 and p-MeCP2 were determined by western blotting in HT115 (**g**) and HCT-8 (**h**) cells following treatments with 6 μg/ml 5-FU or 5 μM KN-93 or both for 24 h. *p < 0.05, **p < 0.01, ***p < 0.001

One of the best characterized calcium responsive regulator on RNA splicing turned out to be an epigenetic factor MeCP2, a multi-talented modulator of chromatin architecture. MeCP2 was reported to be enriched at a specific fraction of alternative exons of genome and enhanced their inclusion [21]. MeCP2 phosphorylation on Serine 421, controlled by nuclear calcium signaling, regulated the off/on state switching of MeCP2 binding to methylated DNAs [22]. To investigate whether MeCP2 is required for *opn* pre-mRNA alternative splicing, MeCP2 targeted siRNAs were transfected into HT115 and HCT-8 cells. Knocking down of MeCP2 increased the production of OPNb and OPNc (Fig. 5a, b). When the cells were treated with 5-AZA for the suppression of DNA methylation, similar increase of OPNb and OPNc were observe in both HT115and HCT-8 cells (Fig. 5c, d). This suggested that the enhanced alternative splicing of the *opn* gene required reduced binding of MeCP2 to DNA at methylated sites.

Moreover, the increases of OPNb and OPNc upon 5-FU treatment were significantly reduced by the inhibition of CaMKII following the treatment of KN-93 (Fig. 5e, f), which could be due to the subsequent inhibition of the phosphorylation activation of MeCP2 at S421 site (Fig. 5g, h). Therefore, MeCP2 was able to control the splicing of OPNb and OPNc, which could be regulated by calcium signaling and DNA methylation.

### MeCP2 binding at *opn* exon4 and exon5 genomic regions was defined upon calcium activation and DNA methylation

**Fig. 6:**
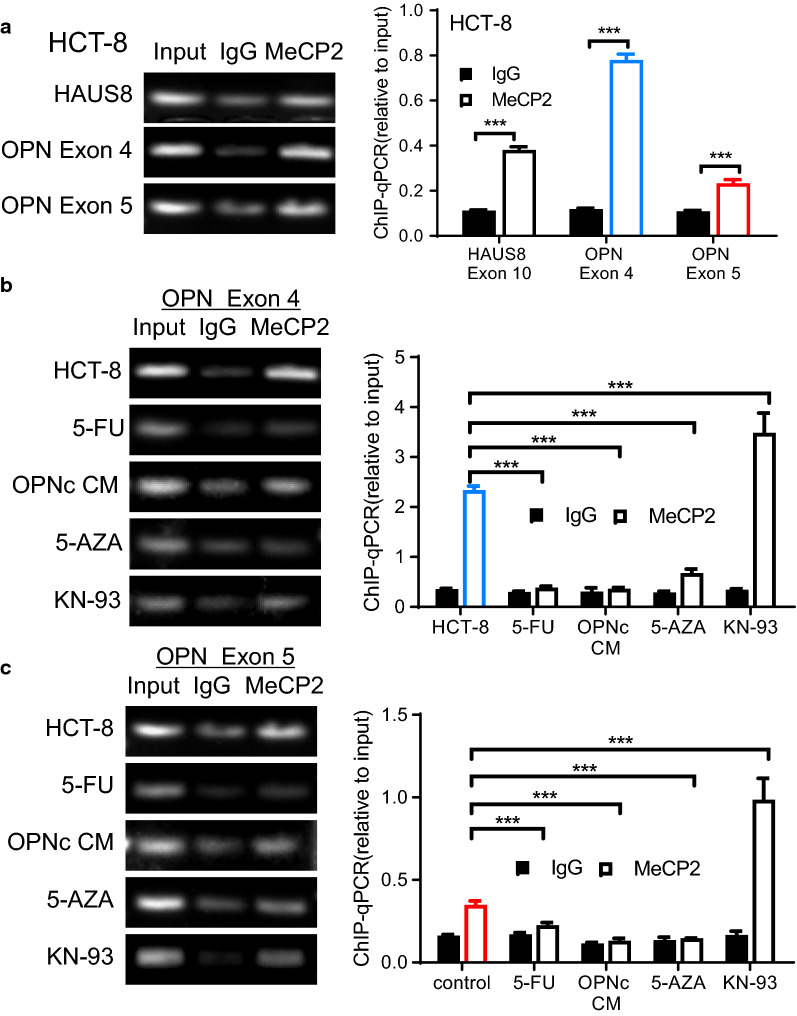
Determinization of MeCP2 binding to *opn* exon 4 and exon 5 by ChIP assays. **a** The binding of MeCP2 to alternatively spliced exon 4 and exon 5 of *opn* gene in non-treated HCT-8 cells, shown in agarose gel electrophoresis (left) and qPCR (right) results. The exon 10 of *HAUS8* gene was used as a positive reference of MeCP2 association, and a mixture of non-specific IgG was used as the assay control. **b** MeCP2 binding to *opn* exon 4 determined by ChIP assays from HCT-8 cells treated with 6 μg/ml 5-FU, OPNc-CM, 5 μg/ml 5-AZA or 5 μM KN-93 for 24 h. **c** MeCP2 binding to *opn* exon 5 under conditions as in (**b**). ***p < 0.001

To find out whether MeCP2 indeed functioned precisely at the *opn* gene alternative splicing exons, we carried out ChIP-qPCR experiments. Fortunately, Maunakea et al. previously analyzed the genome-wide distribution of MeCP2 in colon cancer cell line HCT116 (2013, GSE47678) by ChIP-seq, which helped us to avoid the problem of screening primers to identify the exact target DNA fragments for PCR detections (Additional file 1: Fig. S2).Using *HAUS8* gene as a MeCP2 positive control [23], the results identified the binding of MeCP2 to *opn* exon4 and exon5 in HCT-8 cells at native states (Fig. 6a). When treated with 5-FU or OPNc-CM, the binding of MeCP2 to *opn* exon4 (Fig. 6b) and exon5 (Fig. 6c) were significantly reduced. We then tested whether methylation of *opn* gene would affect the binding of MeCP2 by pretreat HCT-8 cells with 5-AZA. These data showed that DNA hypomethylation resulted in significantly decreased binding of MeCP2 to *opn* exon4 and exon5 by 30% and 43% (Fig. 6b, c). When cells treated with KN-93 to inhibit CaMKII and subsequent phosphorylation MeCP2 at S421, we found that the binding of MeCP2 to *opn* exon4 and exon5 was increased by 1.5 and 2.8 fold (Fig. 6b, c). We concluded that the alternative splicing of *opn* was under controls of MeCP2 binding and was modulated with the S421 phosphorylation of MeCP2 in response to calcium signals.

### MeCP2 S421 phosphorylation correlated to the upregulation of OPNc expression in CRC tissue samples

**Fig. 7:**
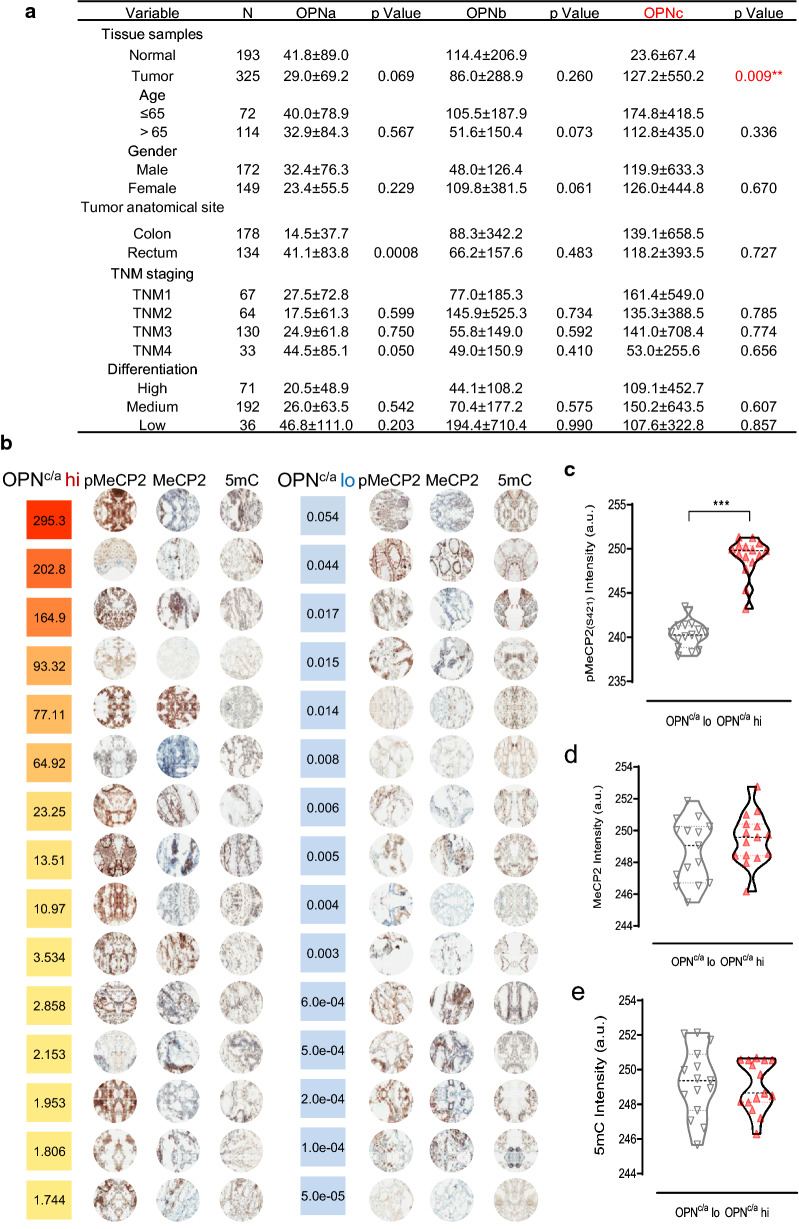
Evaluation of OPNc expression associated with MeCP2 protein phosphorylation in clinical colorectal cancer tissues. **a** Relative mRNA levels of OPN splicing isoforms in colon cancer tissues (n = 325) and the adjacent tissues (n = 193). **b** Immunohistochemistry for p-MeCP2(S421), MeCP2 and 5mC in tissue sections selected from cancer samples of low or high ratios in OPNc/OPNa from RT-qPCR measures (n = 15). **c**, **d** Quantified staining intensity of p-MeCP2 and MeCP2 levels from (**b**). **e** Statistics on DNA methylation by 5-mC staining in OPNc/OPNa high or low groups. **p < 0.01, ***p < 0.001

OPN was previously reported to be at high levels in colorectal cancer tissues than in normal tissues [24], but the data to differentiate the levels of OPN-SIs were very few. Using RT-qPCR with specific primers probing for splicing junctions, we quantified the transcripts of OPNa, OPNb and OPNc for their abundance in the colorectal cancer tissue and the paired paracancerous tissue samples. The results showed high levels of OPNc in cancers were most significantly different than of OPNa and OPNb (Fig. 7a). In evaluate whether the OPNc levels were in relation to MeCP2 phosphorylation and genomic DNA methylation, immunohistochemistry was performed to detect MeCP2, p-MeCP2 and 5mC (5-hydroxymethylcytosine) in selected tissue sections. We ranked the ratio of OPNc to OPNa transcript abundance from high to low based on qPCR quantification of all 325 cancer samples, each 15 samples from the top and bottom were selected for semiquantitative analyses. From the compiled panel (Fig. 7b), positive staining of p-MeCP2 appeared to be more frequent observed in samples with high ratio of OPNc/OPNa, and passed the significance threshold in statistics (Fig. 7c). The staining of MeCP2 and the level of DNA methylation did not show significance compared to the low OPNc/OPNa groups (Fig. 7d, e).

## Discussion

The resistance to anticancer drugs is a major obstacle in cancer clinical practice. Recent studies demonstrated that cancer cell-secreted molecules were functionally involved in conferring chemoresistance by altering TME [1]. From the present study, OPNc, a minor splicing isoform of OPN, seemed to be a fast and sensitive factor upregulated in cells under the cytotoxic pressure (Fig. 1). The secreted OPNc played a dramatically role to promote cell survival as cells were exposed under conditions of drug induced cytotoxic pressure (Fig. 2). Researches have demonstrated the various genetic and epigenetic modifications serves as fundamental natural responses of cells to cope with environmental changes, and will result in a pool of selected cells with genomic alterations and differences in proliferative phenotypes [25, 26]. It can also be inferred that stimulated tumor cells with acquired growth potentials might secrete molecules and spread them out as important signals to synchronize adjacent cells for grouped community response against environmental stress. Among these secreted factors, the splicing isoforms are a good source of candidates, which can be generated rapidly from existing pre-mRNA and accumulate to reach an abundance to mediated and transmit responsive signals in TME. We believe that alternative splicing isoforms, especially to result in secretory proteins, could be effective indicators of chemoresistance and might be of importance as potential targets for the improvement of chemotherapy.

In most cases, to accurately monitor and profile the components of their changes in TME can be extremely challenging due to their complexity. Alternatively, assays to trace cellular signal molecules and the downstream effects in connection with the environmental stimulation can be adopted, especially to determine the dynamics of rapid responses. As a critical second messenger of cells, calcium is an important molecule that responds to environmental stimulation and also links to OPN molecular functions. It was recently recognized that cancer cell released factors could directly trigger a transmissible endoplasmic reticulum stress (TERS) and induced a de novo endoplasmic reticulum stress (ERS) in surrounding recipient cells. Such horizontal transmission of stress signals were observed to enhance the cell resistance to nutrient starvation or exposure in common chemo-drugs [27]. The occurrence of ERS increased the intracellular calcium ([Ca^2+^]i) levels, which further led to the increase of nuclear [Ca^2+^] via the connected structures between endoplasmic reticulum (ER) and nuclear membrane. We observed robust responses of nuclear calcium increase when cells were treated with the OPN-CMs, especially OPNc and OPNb enriched CMs (Fig. 3). Our study emphasized that the nuclear calcium signal could be considered as a rapid and sensitive indicator to character the response of cells to environmental stimulation in conditioned microenvironment. The treatment with KN-93, the specific CaMKII inhibitor, significantly attenuated the OPN-CMs increased cell survivals from the 5-FU administration (Fig. 4).The results also implied for tumor progression linked to the activation of calcium signal pathways and the activity of CaMK needed to be considered.

Alternative splicing (AS) contributes greatly to the proteomic complexity. How AS is regulated by environmental stimuli to sculpt cellular phenotypic properties, particularly in highly heterogeneous and progressing malignant tumors, is yet a poor understood area of emerging interest from both laboratory research and clinical sides. Recent studies in cardiomyocytes, neurons and endocrine cells have begun to shed light on the regulation of calcium signals on pre-mRNA alternative splicing [28, 29]. Our results demonstrated that the generation of OPNc (Fig. 5) was a nuclear calcium-controlled event involving the MeCP2 phosphorylation at position serine 421, and was dependent to DNA methylation for binding MeCP2 to the *opn* alternative splicing exons (Fig. 6). This finding was also supported by the fact where p-MeCP2 was more intensive in colon cancer tissues with elevated mRNA levels of OPNc (Fig. 7). These results were in support of the recently developed understanding that alternative splicing events were largely subjected to the controls of epigenetic regulations [30].

Epigenetic modifications at the genome in a natural mechanism of cells to adapt to microenvironment and functions as an immediate response of cells to environmental stimulation. Besides from numerous mutations, environmental selection from epigenetic changes during tumor progression may also occur. Subtle changes in chromatin configuration are sufficient to trigger the abnormal splicing of pre-mRNA and cause the accumulation of minor products in the nuclei. Although the detailed mechanisms regarding the interplay between environmental factors and epigenetic genome modifications remain elusive, especially in cancers, however, it is increasingly recognized that nuclear Ca^2+^ could be important for the generation of specific splicing isoform. This notion was supported by our experiments with OPN-SIs and will help to explain how the transmit and spread of stress signals can form a positive feedback loop among cells in TME. An apparent limitation of the present study was that we were not able to assess the proposed regulation mechanism of OPN splicing for long-term outcomes. Our results focused on demonstrating that, as an example, the production of specific extracellular splicing isoform not only could indicate the changes in epigenetic modifications, but also could be used as target molecule to alter cell phenotypes or cell fate.

## Conclusions

In conclusion, our present study out lined a mechanism by which tumor cells under 5-FU treatment could stimulate adjacent cells by spreading secretory OPNc to survive from drug-induced microenvironmental stress. The increased production of OPNc involved an epigenetic regulation of MeCP2 at S421 phosphorylation, which reduced its binding to *opn* gene methylated alternative exons. The activation of nuclear calcium signals and activities of CaMKII seemed to be required during the regulatory process. Our findings indicated that the role of OPNc in TME might of great importance during the development of chemoresistance. It also implied that inhibiting OPN splicing or other tumor specific splicing through the epigenetic modulator agent, in combination with conventional chemotherapies, could present as an effective approach to preventing tumor progression and recurrence.

## Supplementary information

**Additional file 1:** **Figure S1.** Western blot of OPN proteins in CMs from OPN-SIs overexpressing cells. Cell lysate of transfected HT115 (**a**) or HCT-8cells (**b**). CMs collected from transfected HT115 (**c**) or HCT-8cells (**d**). **Figure S2.** ChIP-Seq dataset (GSE47678, 2013) from the GEO database revealed MeCP2 interaction sites in peaks at theopngene exons 4 and exon 5 regions in HCT116 colorectal cancer cells.

## Data Availability

Public online or upon request.
