# Supplementary material for: Epigenetic regulation of osteopontin splicing isoform c defines its role as a microenvironmental factor to promote the survival of colon cancer cells from 5-FU treatment
Source: Cancer Cell Int. 2020 Sep 14;20:452. doi: 10.1186/s12935-020-01541-z (PMC7491101; doi:10.1186/s12935-020-01541-z)
Supplement: Supplementary file 1 — Additional file 1: Figure S1. Western blot of OPN proteins in CMs from OPN-SIs overexpressing cells. Cell lysate of transfected HT115 (a) or HCT-8cells (b). CMs collected from transfected HT115 (c) or HCT-8cells (d). Figure S2. ChIP-Seq dataset (GSE47678, 2013) from the GEO database revealed MeCP2 interaction sites in peaks at theopngene exons 4 and exon 5 regions in HCT116 colorectal cancer cells. [file 12935_2020_1541_MOESM1_ESM.docx]

**Additional information**

**Materials and methods**

**Western blotting**

Assays was performed as earlier described [1]. Briefly, the cell lysates were collected and subjected to 10% SDS-polyacrylamide gel electrophoresis (SDS-PAGE). The separated proteins were transferred onto polyvinylidene fluoride (PVDF) filters. The probing antibodies used were as against the following antigens: OPN (8448, rabbit polyclonal antibody, 1:1000, abcam, Cambridge, MA, USA), Flag (2044, rabbit monoclonal antibody, 1:1000, Cell Signaling Technology, Danvers, MA, USA) and GAPDH (TA-08, mouse monoclonal antibody, 1:3000, ZSGB-BIO, Beijing, China). Horseradish peroxidase-conjugated secondary antibodies were obtained from ZSGB-BIO (Beijing, China). Finally, the blots were visualized via enzyme linked chemiluminescence using the ECL kit (NCM Biotech).

**Figure S1.** Western blot of OPN proteins in CMs from OPN-SIs overexpressing cells. **a**, **b** Cell lysate of transfected HT115 (**a**) or HCT-8 cells (**b**). **c**, **d** CMs collected from transfected HT115 (**c**) or HCT-8 cells (**d**).

**Figure S2.** ChIP-Seq dataset (GSE47678, 2013) from the GEO database revealed MeCP2 interaction sites in peaks at the *opn* gene exons 4 and exon 5 regions in HCT116 colorectal cancer cells.

**References**

1. Hao C, Cui Y, Hu MU et al. OPN-a splicing variant expression in non-small cell lung cancer and its effects on the bone metastatic abilities of lung cancer cells in vitro. Anticancer Res. 2017; 37(5)**:** 2245-54.
